# Supplementary material for: A distal regulatory region of a class I human histone deacetylase
Source: Nat Commun. 2020 Jul 31;11:3841. doi: 10.1038/s41467-020-17610-w (PMC7395746; doi:10.1038/s41467-020-17610-w)
Supplement: Supplementary file 1 — Supplementary Information [file 41467_2020_17610_MOESM1_ESM.pdf]

Supplementary Information

A Distal Regulatory Region of a Class I Human Histone Deacetylase

Nicolas D. Werbeck, Vaibhav Kumar Shukla, et al.

Supplementary Table 1: Extracted values of $\sqrt{p_m(1-p_m)}|\Delta\varpi_C|$ and associated uncertainties obtained from fits of relaxation dispersion data recorded on free HDAC8

Residue	$\sqrt{p_m(1-p_m)}|\Delta\varpi_C|$ (ppm)	Error (s.d.)
M27 C^{ε}	0.108	0.007
I34 $C^{\delta1}$	0.068	0.009
M40 C^{ε}	0.009	0.003
I45 $C^{\delta1}$	0.000	0.052
I56 $C^{\delta1}$	0.033	0.013
I94 $C^{\delta1}$	0.028	0.019
I231 $C^{\delta1}$	0.022	0.020
I284 $C^{\delta1}$	0.056	0.010
I300 $C^{\delta1}$	0.047	0.010
I362 $C^{\delta1}$	0.019	0.023

Supplementary Table 2: Extracted values of $|\Delta\varpi_C|$ and associated uncertainties obtained from fits of relaxation dispersion data recorded on TSA-bound HDAC8

Residue	$\|\Delta\varpi_C\|$ (ppm)	Error
I19 $C^{\delta1}$	0.087	0.091
M27 C^{ε}	1.353	0.156
M40 C^{ε}	0.626	0.085
I45 $C^{\delta1}$	0.200	0.043
I56 $C^{\delta1}$	0.326	0.046
I94 $C^{\delta1}$	0.327	0.054
I127 $C^{\delta1}$	0.025	0.246
I135 $C^{\delta1}$	0.081	0.112
I231 $C^{\delta1}$	0.078	0.104
I269 $C^{\delta1}$	0.141	0.067
I284 $C^{\delta1}$	0.176	0.048
I300 $C^{\delta1}$	0.176	0.055
I322 $C^{\delta1}$	0.080	0.129
I331 $C^{\delta1}$	0.081	0.097
I362 $C^{\delta1}$	0.117	0.054
I365 $C^{\delta1}$	0.079	0.077
I369 $C^{\delta1}$	0.039	0.222

Supplementary Table 3: List of primers used for introducing point mutations

Mutant Name	Primer sequence
I19V#	FP: 5`-CGCTGGTCCCGGTTTATGTGTATAGTCCCGAGTATG-3`<br>RP: 5`-CATACTCGGGACTATACACATAAACCGGGACCAGCG-3`
I34V#	FP: 5`-GTGACTCCCTGGCCAAGGTGCCCAAACGGGCCAGTATG-3`<br>RP: 5`-CATACTGGCCCGTTTGGGCACCTTGGCCAGGGAGTCAC-3`
I45V#	FP: 5`-GTATGGTGCATTCTTTGGTGGAAGCGTATGCACTGC-3`<br>RP: 5`-GCAGTGCATACGCTTCCACCAAAGAATGCACCATAC-3`
I56V#	FP: 5`-GCATAAGCAGATGAGGGTGGTTAAGCCTAAAGTGGC-3`<br>RP: 5`-GCCACTTTAGGCTTAACCACCCTCATC1TGCTTATGC-3`
I94V#	FP: 5`-GATGATCATCCGGACTCCGTGGAATATGGGCTAGGTTATG-3'<br>RP: 5`-CATAACCTAGCCCATATTCCACGGAGTCCGGATGATCATC-3'
I108V#	FP: 5`- CCACTGAAGGCGTATTTGACTATGCAGCAGCTATAGGAG-3`<br>RP: 5`-CATAGTCAAATACGCCTTCAGTGGCTGGGCAGTCATAACC-3`
I115V#	FP: 5`- GACTATGCAGCAGCTGTGGGAGGGGCTACGATC-3`<br>RP: 5`-GATCGTAGCCCCTCCCACAGCTGCTGCATAGTC-3`
I120V#	FP: 5`-CTATAGGAGGGGCTACGGTGACAGCTGCCCAATGCC-3`<br>RP: 5`-GGCATTGGGCAGCTGTCACCGTAGCCCCTCCTATAG-3`
I127V#	FP: 5`-GCTGCCCAATGCCTGGTGGACGGAATGTGCAAAG-3'<br>RP: 5`-CTTTGCACATTCCGTCCACCAGGCATTGGGCAGC-3'
I135V#	FP: 5`-GAATGTGCAAAGTAGCAGTGAACTGGTCTGGAGGGTG-3'<br>RP: 5`-CACCCTCCAGACCAGTTCACTGCTACTTTGCACATTC-3'
I162V#	FP: 5`-CTGTCCTGGGAGTATTACGATTGCGACGGAAATTTGAGC-3'<br>RP: 5`-TCCGTCGCAATCGTAATACTCCCAGGACAGCATCATTGAG-3'
I172V#	FP: 5`- GACGGAAATTTGAGCGTGTGCTCTACGTGGATTTGG-3`<br>RP: 5`-CCAAATCCACGTAGAGCACACGCTCAAATTTCCGTC-3`
I231V#	FP: 5`-CTACAGTGTAAATGTGCCCGTGCAGGATGGCATACAAGATG-3'<br>RP: 5`-CATCTTGTATGCCATCCTGCACGGGCACATTTACACTGTAG-3'
I235V#	FP: 5`-GTGCCCATTCAGGATGGCGTGCAAGATGAAAAATATTACC-3`<br>RP: 5`-GGTAATATTTTTCATCTTGCACGCCATCCTGAATGGGCAC-3`
I243V#	FP: 5`-GATGAAAAATATTACCAGATCTGTGAAAGTGTACTAAAGG-3`<br>RP: 5`-CCTTTAGTACACTTTCACAGATCTGGTAATATTTTTCATC-3`
I269V#	FP: 5`-CTGGGAGCTGACACAGTGGCTGGGGATCCCATG-3`<br>RP: 5`- CATGGGATCCCCAGCCACTGTGTCAGCTCCCAG-3`
I284V#	FP: 5`-CATGACTCCAGTGGGAGTGGGCAAGTGTCTTAAG-3`<br>RP: 5`-CTTAAGACACTTGCCCACTCCCACTGGAGTCATG-3`
I291V#	FP: 5`-GCAAGTGTCTTAAGTACGTGCTTCAATGGCAGTTGGC-3`<br>RP: 5`-GCCAACTGCCATTGAAGCACGTACTTAAGACACTTGC-3`
I300V#	FP: 5`-CAGTTGGCAACACTCGTGTTGGGAGGAGGAGG-3`<br>RP: 5`- CCTCCTCCTCCCAACACGAGTGTTGCCAACTG-3`
I322V#	FP: 5`-CATACTTGACCGGGGTCGTGCTAGGGAAAACACTATC-3`<br>RP: 5`-GATAGTGTTTTCCCTAGCACGACCCCGGTCAAGTATG-3`
I331V#	FP: 5`-GAAAACACTATCCTCTGAGGTGCCAGATCATGAGTTTTTCAC-3`<br>RP: 5`-GTGAAAAACTCATGATCTGGCACCTCAGAGGATAGTGTTTTC-3`
I348V#	FP: 5`-GATTATGTCCTGGAAGTGACGCCAAGCTGCCGG-3`<br>RP: 5`-CCGGCAGCTTGGCGTCACTTCCAGGACATAATC-3`
I362V#	FP: 5`- GCAATGAGCCCCACCGAGTGCAACAAATCCTCAACTAC-3`<br>RP: 5`-GTAGTTGAGGATTTGTTGCACTCGGTGGGGCTCATTGC-3`
I365V#	FP: 5`-CCCACCGAATCCAACAAGTGCTCAACTACATCAAAGG-3`<br>RP: 5`-CCTTTGATGTAGTTGAGCACTTGTTGGATTCGGTGGG-3`
I369V#	FP: 5`-CCAACAAATCCTCAACTACGTGAAAGGGAATCTGAAGCATG-3`<br>RP: 5`-CATGCTTCAGATTCCCTTTCACGTAGTTGAGGATTTGTTGG-3`
I19A#	FP: 5`-TCGCTGGTCCCGGTTTATGCCTATAGTCCCGAG-3`<br>RP: 5`-ACACATACTGACATACTCGGGACTATAGGCATAAACCGGGAC-3`

I19S#	FP: 5`-TCGCTGGTCCCGGTTTATTCCTATAGTCCCGAGTATGTC-3`<br>RP: 5`-GACATACTCGGGACTATAGGAATAAACCGGGACCAGCGA-3`
I19A*	FP: 5`-TCGCTGGTTCCAGTCTATGCTTACTCACCGGAATATGTT-3`<br>RP: 5`-AACATATTCCGGTGAGTAAGCATAGACTGGAACCAGCGA-3`
I19S*	FP: 5`-TCGCTGGTTCCAGTCTATAGTTACTCACCGGAATATGTT-3`<br>RP: 5`-AACATATTCCGGTGAGTAACTATAGACTGGAACCAGCGA-3`
S39E*	FP: 5`- CCAAACGGGCCGAAATGGTGCATTCTTTGATTG-3`<br>RP: 5`- CAATCAAAGAATGCACCATTTCGGCCCGTTTGG-3`
M27A*	FP: 5'-CCGGAATATGTTAGTGCGTGCGATTCCCTGG-3'<br>RP: 5'-CCAGGGAATCGCACGCACTAACATATTCCGG-3'
M40A*	FP: 5`- CGAAACGTGCCTCAGCGGTTCATTCGCTG-3'<br>RP: 5`- CAGCGAATGAACCGCTGAGGCACGTTTCG-3'
S39EM40A#	FP: 5`-AAGATACCCAAACGGGCCGAAGCGGTGCATTC-3`<br>RP: 5`-TCAATCAAAGAATGCACCGCTTCGGCCCGTTT-3`
F336A#	FP: 5`-CCTCTGAGATCCCAGATCATGAGGCTTTCACAGCGTAC-3`<br>RP: 5`-ATCAGGACCGTACGCTGTGAAAGCCTCATGATCTGG-3`
F336A*	FP: 5`- ATTCCGGACCATGAAGCTTTCACCGCGTATGGC-3`<br>RP: 5`-GCCATACGCGGTGAAAGCTTCATGGTCCGGAAT-3`

#) Primer for the construct in the pET21b expression vector, with a C-terminal hexa-histidine tag.
*) Primer for Codon optimised DNA sequence used in in pET29b+ having TEV-cleavable N-terminal $(His)_6$-NusA tag

Codon optimised DNA sequence for HDAC8 used in pET29b+ expression vector with TEV-cleavable N-terminal $(His)_6$-NusA tag

ATGGAAGAACCGGAAGAACCGGCGGATTCAGGCCAATCGCTGGTTCCGGTCTATATTTAC
TCACCGGAATATGTTAGTATGTGCGATTCCCTGGCGAAAATCCCGAAACGTGCCTCAATG
GTTCATTCGCTGATTGAAGCATACGCTCTGCACAAACAGATGCGCATCGTCAAACCGAAA
GTGGCAAGCATGGAAGAAATGGCTACCTTTCATACGGATGCGTATCTGCAGCACCTGCAA
AAAGTGAGCCAGGAAGGTGATGACGATCATCCGGATTCTATTGAATATGGCCTGGGTTAC
GACTGCCCGGCAACCGAAGGCATCTTCGATTACGCTGCGGCCATCGGCGGTGCGACCATT
ACGGCAGCTCAGTGCCTGATCGATGGCATGTGTAAAGTGGCCATTAACTGGTCAGGCGGT
TGGCATCACGCGAAAAAAGACGAAGCCTCGGGTTTTTGTTATCTGAATGATGCGGTTCTG
GGCATCCTGCGTCTGCGTCGCAAATTCGAACGCATTCTGTACGTGGATCTGGACCTGCAT
CACGGCGATGGTGTTGAAGACGCGTTTAGCTTCACCTCTAAAGTCATGACCGTGAGTCTG
CACAAATTTTCCCCGGGCTTTTTCCCGGGCACCGGTGATGTTAGTGACGTCGGCCTGGGTA
AAGGCCGTTATTACTCCGTGAACGTTCCGATTCAAGATGGCATCCAGGACGAAAAATATT
ACCAGATTTGTGAAAGCGTTCTGAAAGAAGTCTATCAGGCATTTAACCCGAAAGCTGTCG
TGCTGCAACTGGGTGCCGATACCATTGCGGGTGACCCGATGTGCTCTTTCAATATGACGC
CGGTGGGTATCGGCAAATGTCTGAAATATATTCTGCAGTGGCAACTGGCCACCCTGATTC
TGGGCGGTGGCGGTTATAACCTGGCGAATACCGCACGTTGCTGGACCTACCTGACCGGTG
TGATCCTGGGCAAAACCCTGAGCTCTGAAATTCCGGACCATGAATTTTTCACCGCGTATG
GCCCGGATTACGTTCTGGAAATTACGCCGAGCTGTCGTCCGGATCGCAATGAACCGCACC
GCATCCAACAAATCCTGAACTACATCAAAGGTAATCTGAAACACGTCGTCTAATAA

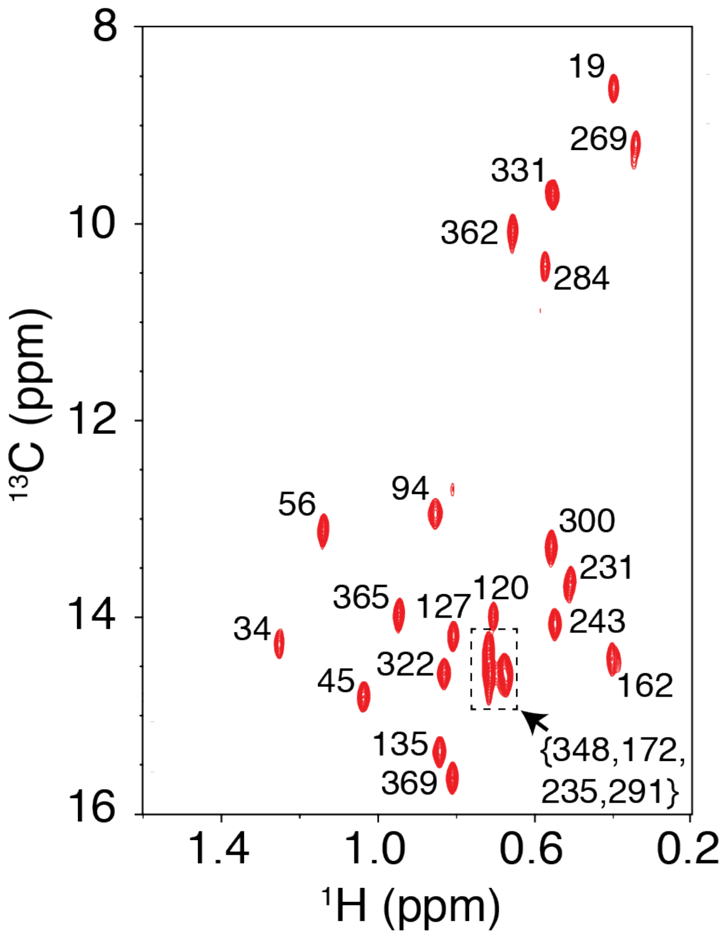

Supplementary Figure 1 | Assigned isoleucine δ1-methyl-groups of HDAC8. The assignment of 23 out of the 25 isoleucines was obtained (I108 and I115 remain unassigned). The methyl-TROSY spectrum shown is the reference plane (T_{relax}=0 ms) of the multi-quantum CPMG relaxation dispersion experiment recorded at a static magnetic field strength of 22.3 T.

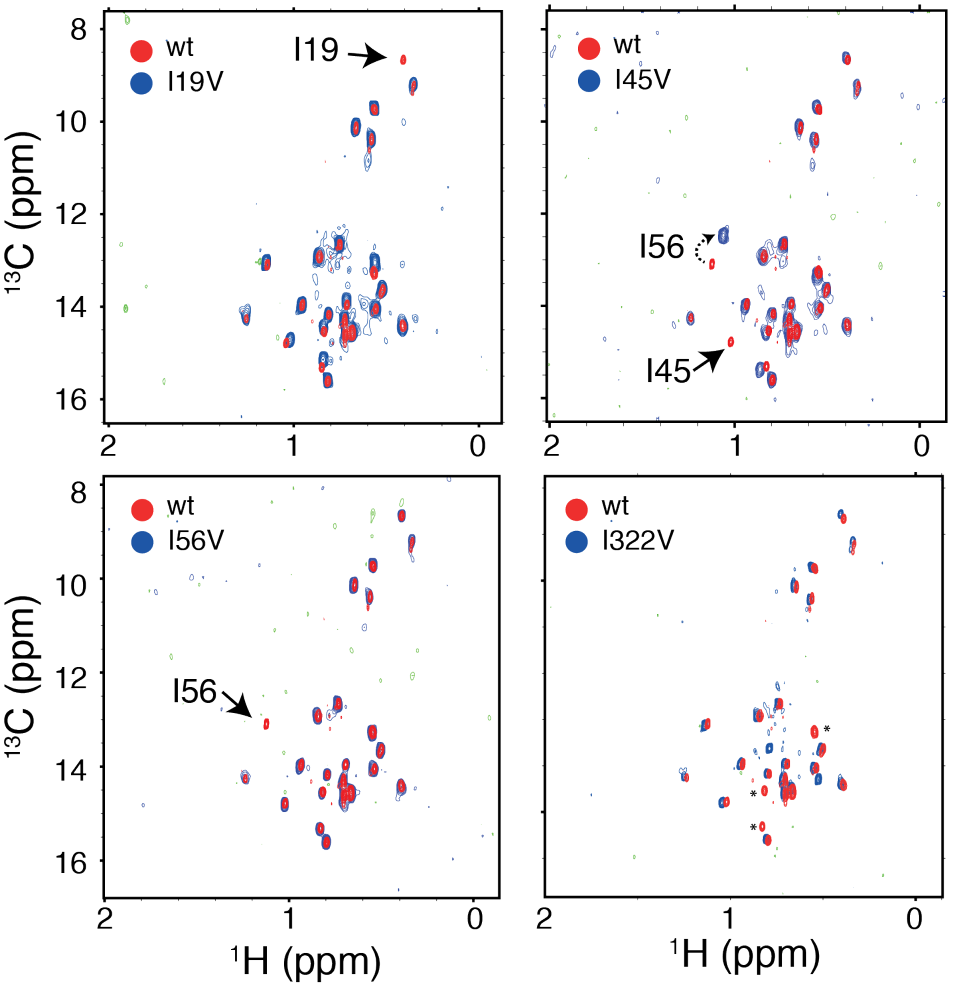

Supplementary Figure 2 | Four examples of isoleucine-to-valine mutations. The mutation I45V leads to changes in the position of I56, further confirming the allosteric network. In contrast, mutation of I56 to valine does not cause a change in the position of I45, presumably due to the flexible nature of the I56 side chains (near-random-coil ^{13}C chemical shift). The mutation I322V is an example where multiple large changes occur in the spectrum (marked with *). All spectra were recorded at a static magnetic field strength of 14.8 T.

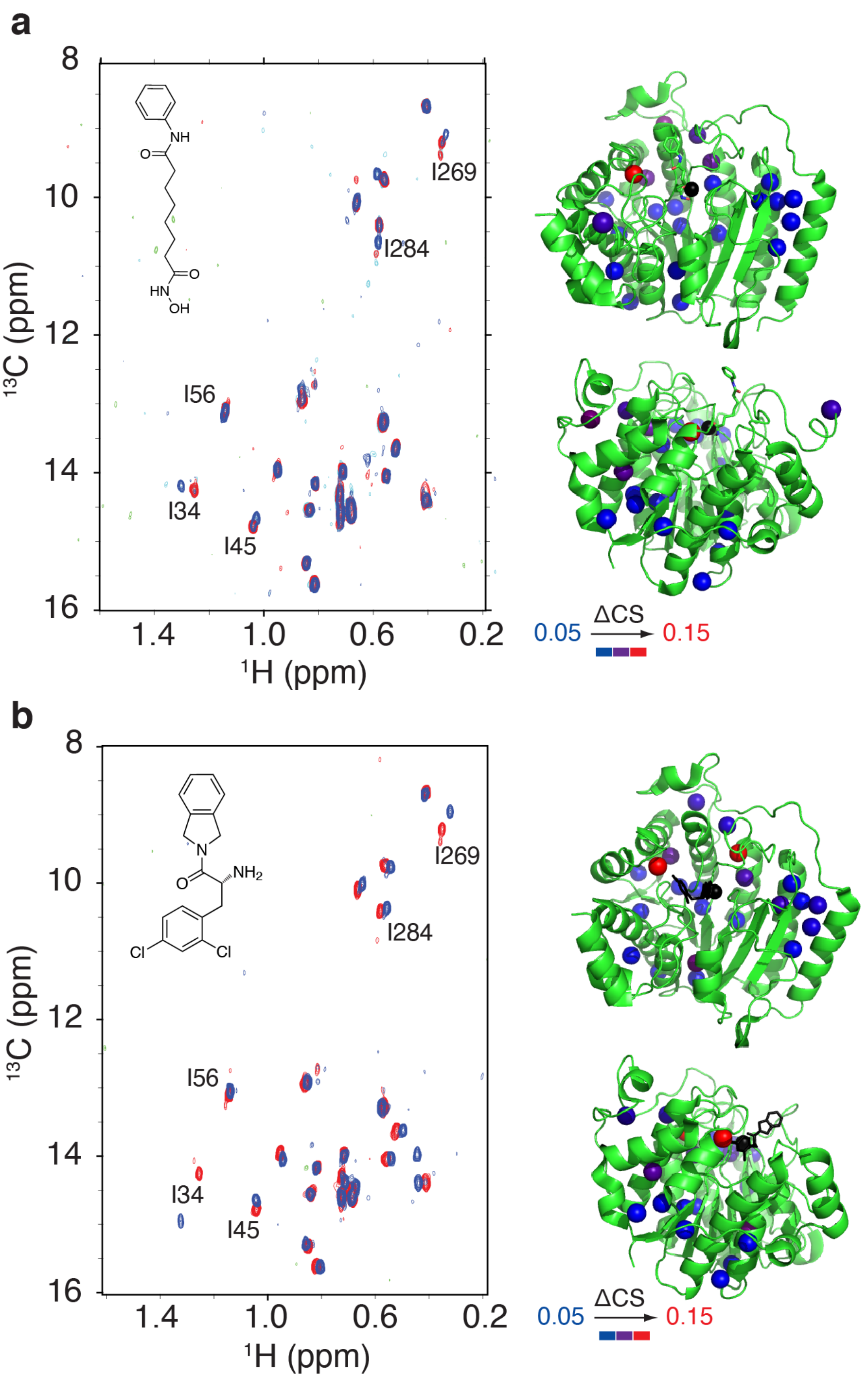

Supplementary Figure 3 | a. Titration of HDAC8 with the non-selective hydroxamic acid HDAC inhibitor SAHA. (red) Free HDAC8, (blue) HDAC8 (30 µM) in complex with SAHA (83 µM). The hydroxamic acid (HO-NH-CO-R) is the zinc-binding motif. **b.** Titration of HDAC8 with the selective HDAC8 inhibitor (*R*)-2-amino-3-(2,4-dichlorophenyl)-1-(1,3-dihydroisoindol-2-yl)-propan-1-one (DCPI). (Red) Free HDAC8, (blue) HDAC8 (30 µM) in complex with DCPI (86 µM). The -CO-CH(R)-NH_2- group is the zinc-binding motif.

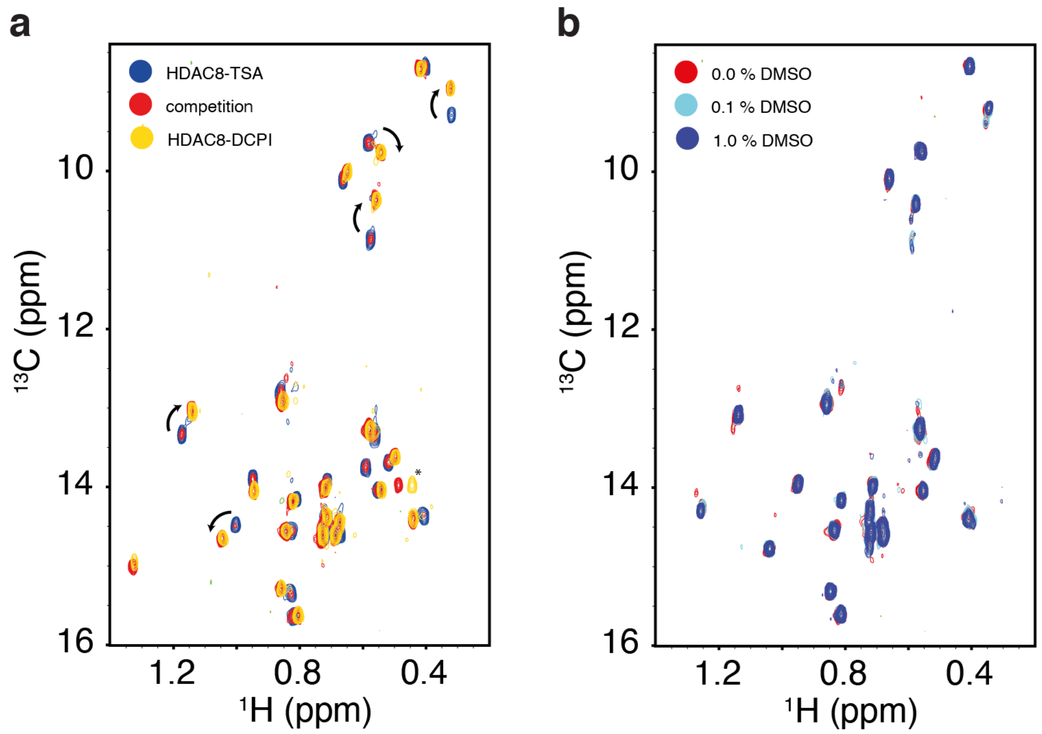

Supplementary Figure 4 | Control experiments: a. TSA competition with the DCPI inhibitor. (Blue) HDAC8 bound to the inhibitor TSA, (yellow) HDAC8 bound to the inhibitor DCPI, (red) HDAC8 (~30 μM) first bound to TSA (~75 μM) and then competed out with the stronger binding DCPI inhibitor (~450 μM). When TSA is competed out, the isoleucine signals shift back towards their original position with DCPI alone, thus showing that the chemical shift changes observed far from the active site upon complex-formation with TSA are not due to unspecific binding. The peak labelled marked by * is unassigned. **b.** Titration of HDAC8 with DMSO. There are only negligible changes in the observed chemical shifts as a function of the concentration of DMSO.

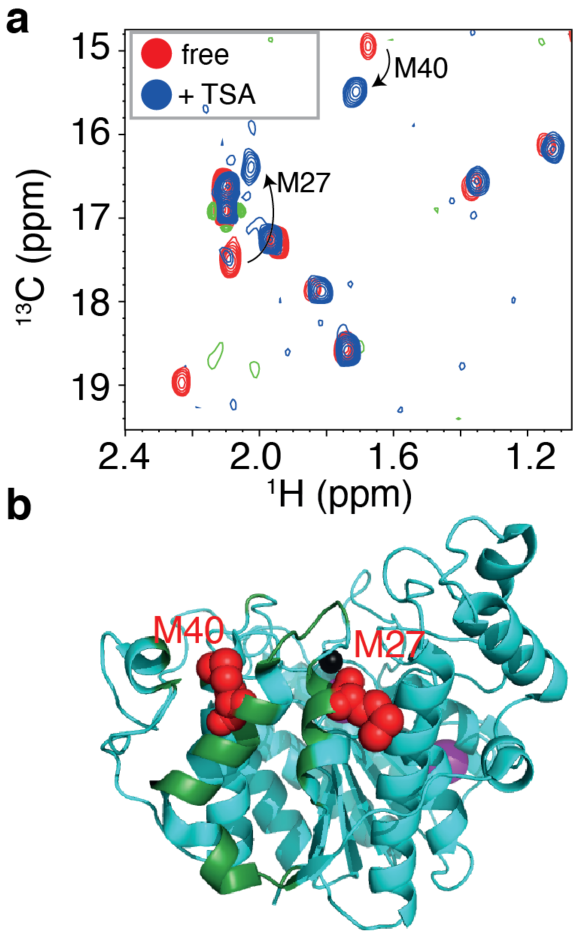

Supplementary Figure 5 | a. HDAC8 methionine cross-peaks before (red) and after (blue) addition of excess TSA. Movements of the two assigned methionine cross-peaks are highlighted. **b.** The two methionine residues, M27 and M40, highlighted on the HDAC8 structure (PDB: 2V5W). Highlighted in green is the helix1-loop1-helix2 motif.

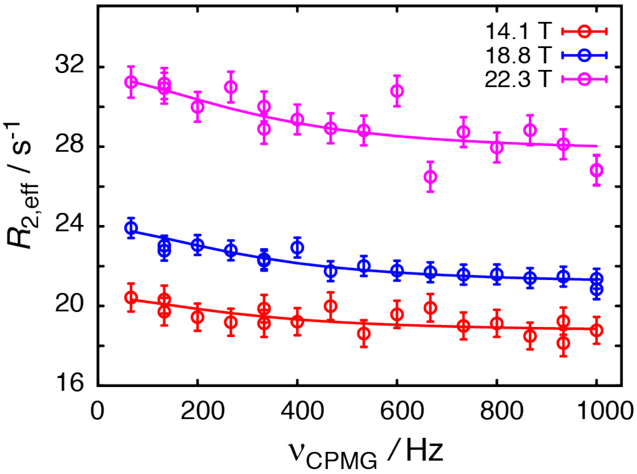

Supplementary Figure 6 | Methyl multiple-quantum relaxation dispersion profiles ($R_{2,\text{eff}}$ *vs.* ν_{CPMG}) of I34 $^{13}C^{\delta1}/^{1}H^{\delta1}$ obtained for ligand-free wild-type HDAC8. Circles represent experimental data, vertical lines represent the uncertainties of the experimental relaxation rates and the solid line is the result of the global least-squares fit to a two-state model. From the least-squared fit it is found that $\sqrt{p_{\text{m}}(1-p_{\text{m}})}|\Delta\varpi_{\text{C}}|$ = 0.068 ppm ± 0.008 ppm.

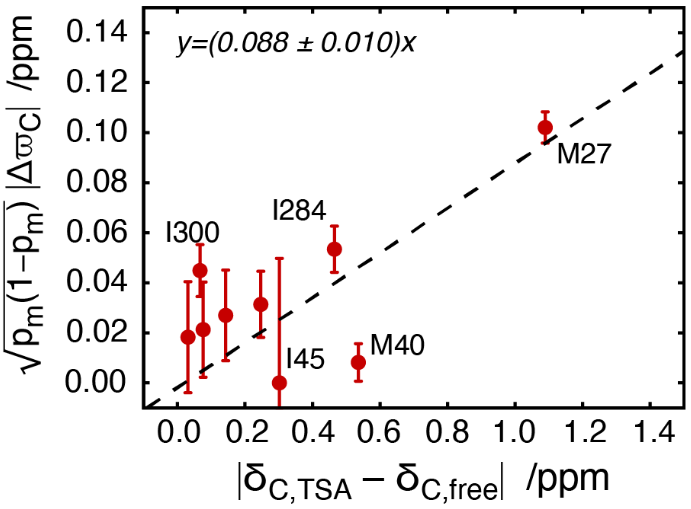

Supplementary Figure 7 | Correlation between ^{13}C chemical shift differences between free HDAC8 and HDAC8 with excess TSA, and $\sqrt{p_m(1-p_m)}|\Delta\varpi_C|$ obtained from CPMG relaxation dispersion experiments on free HDAC8. The slope of 0.088 indicates that the population of the low-populated state is $p_m \sim 0.8\%$. I34 shows relaxation dispersion but this residue is not visible in the TSA-bound state. The dynamics of I34 on the μs–ms timescale is in agreement with computational and structural studies.

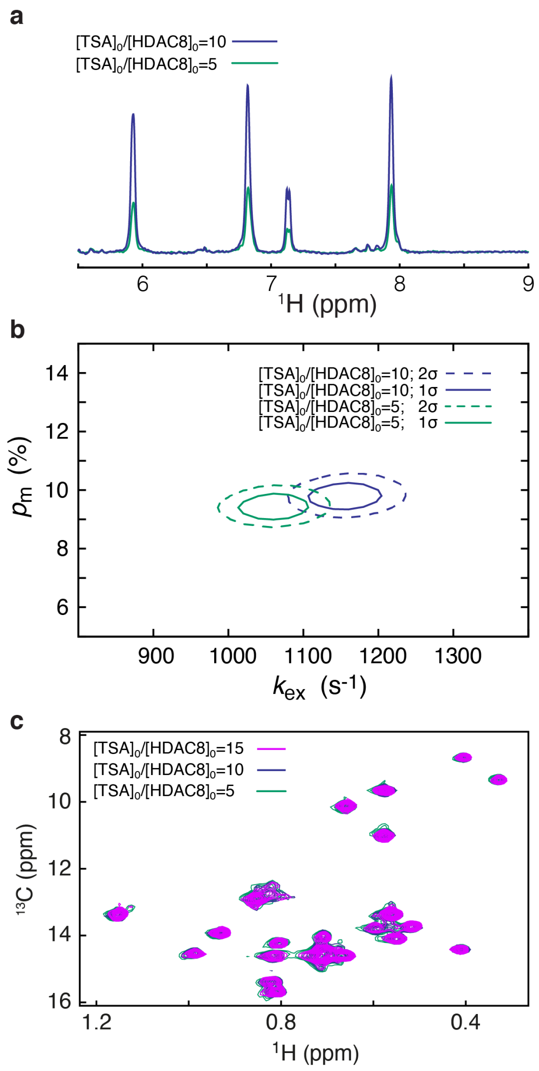

Supplementary Figure 8 | Exchange observed for the HDAC8:TSA report on a unimolecular reaction: CPMG relaxation dispersions were obtained with increasing concentrations of the TSA inhibitor. **a.** One-dimensional ^{1}H NMR control experiment, showing that excess free TSA more than doubles between the two experiments. **b.** Shown is the derived χ^2 surface for samples with HDAC8 and TSA added in ratios of ~1:5 (green) and ~1:10 (blue), respectively. The least-squares fits were performed for fixed values of k_{ex} and p_m over the grid shown to obtain a value for χ^2 at each point. Thus, the minimum of χ^2 at each point was determined by optimizing $\Delta\varpi_C$ for each residue and $R_{2,0}$ for each residue and magnetic field. The contours show the one-standard-deviation (1σ; solid line) and two-standard-deviations (2σ; dashed line) intervals calculated from the obtained χ^2. For a HDAC8:TSA ratio of 1:5, k_{ex} = 1060 ± 33 s^{-1} and p_m = 9.4% ± 0.3%, while for a HDAC8:TSA ratio of 1:10 k_{ex} = 1154 ± 40 s^{-1} and p_m = 9.8% ± 0.4%. Thus, the CPMG relaxation dispersions observed for the TSA-bound form of HDAC8 are reporting on a chemical exchange process independent of the TSA concentration and are thus unimolecular in nature. **c**. Methyl-TROSY spectra obtained for samples with HDAC8 and TSA added in ratios of ~1:5 (green), ~1:10 (blue), and ~1:15 (purple). No chemical shift changes are observed, thereby confirming that potential low-affinity binding of a second TSA molecule to HDAC8 does not affect the NMR results.

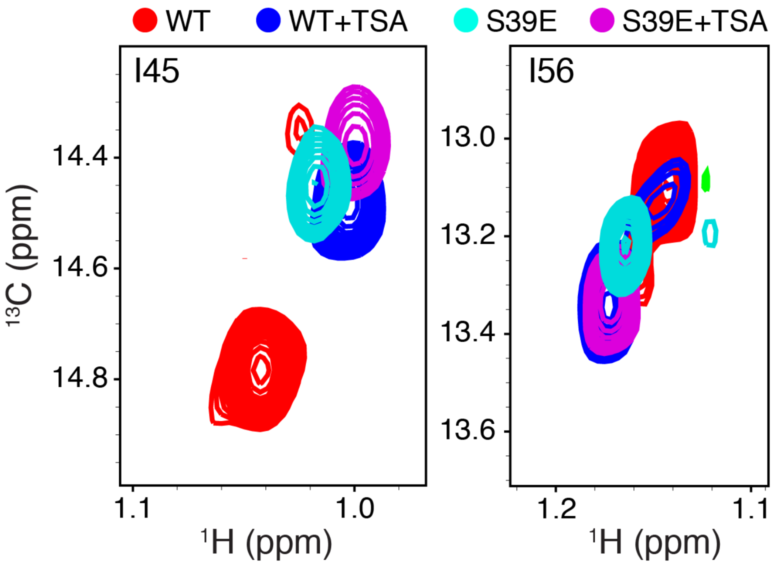

Supplementary Figure 9 | Overlay of methyl-TROSY NMR spectra of I45 and I56 isoleucine δ1-methyl groups of wild-type HDAC8 (red), WT-HDAC8 with 2.5eq TSA (blue), S39E HDAC8 (cyan), and S39E HDAC8 with 2.5eq TSA (purple).

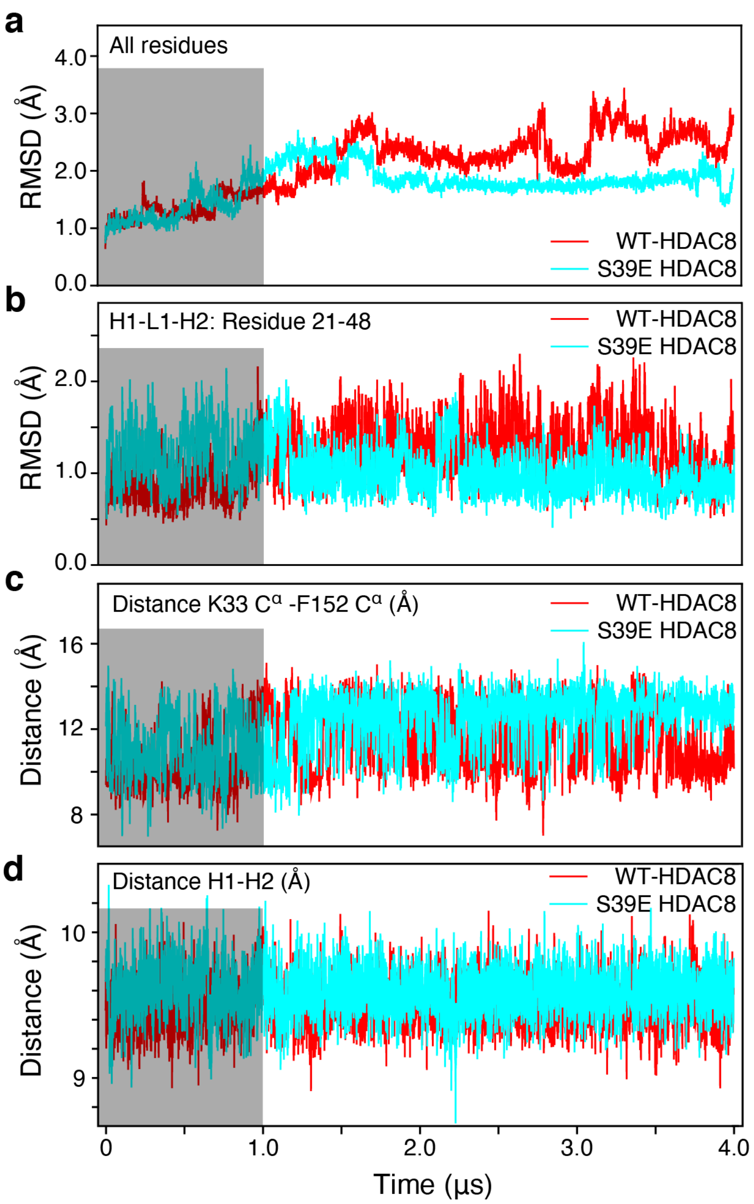

Supplementary Figure 10 | a. and **b.** Root mean square deviations (r.m.s.d.) to the initial structure for molecular dynamics simulations of wild-type HDAC8 (red) and S39E HDAC8 (cyan). **a.** r.m.s.d. calculated for the full structure and **b.** r.m.s.d. calculated for the H1-L1-H2 motif. Prior to calculation of r.m.s.d. the structures were aligned against the backbone. **c**. Distance between K33 C^{α} and F152 C^{α} for the simulation of wild-type HDAC8 (red) and S39E-HDAC8 (cyan) shown as a function of simulation time. **d**. Distance between the centre-of-masses of helix1 (H1) and helix2 (H2) for wild-type HDAC8 (red) and S39E-HDAC8 (cyan) shown as a function of simulation time. The grey areas show the part of the simulation considered as equilibration and thus not included in the analysis.
