# Supplementary material for: A distal regulatory region of a class I human histone deacetylase
Source: Nat Commun. 2020 Jul 31;11:3841. doi: 10.1038/s41467-020-17610-w (PMC7395746; doi:10.1038/s41467-020-17610-w)
Supplement: Supplementary file 3 — Reporting Summary [file 41467_2020_17610_MOESM3_ESM.pdf]

nature research

Corresponding author(s): Prof. D. Flemming Hansen

Last updated by author(s): Jul 2, 2020

Reporting Summary

Nature Research wishes to improve the reproducibility of the work that we publish. This form provides structure for consistency and transparency in reporting. For further information on Nature Research policies, see our Editorial Policies and the Editorial Policy Checklist.

Statistics

For all statistical analyses, confirm that the following items are present in the figure legend, table legend, main text, or Methods section.

n/a	Confirmed	
☐	☒	The exact sample size (*n*) for each experimental group/condition, given as a discrete number and unit of measurement
☐	☒	A statement on whether measurements were taken from distinct samples or whether the same sample was measured repeatedly
☒	☐	The statistical test(s) used AND whether they are one- or two-sided<br>*Only common tests should be described solely by name; describe more complex techniques in the Methods section.*
☐	☒	A description of all covariates tested
☒	☐	A description of any assumptions or corrections, such as tests of normality and adjustment for multiple comparisons
☐	☒	A full description of the statistical parameters including central tendency (e.g. means) or other basic estimates (e.g. regression coefficient) AND variation (e.g. standard deviation) or associated estimates of uncertainty (e.g. confidence intervals)
☒	☐	For null hypothesis testing, the test statistic (e.g. *F*, *t*, *r*) with confidence intervals, effect sizes, degrees of freedom and *P* value noted<br>*Give P values as exact values whenever suitable.*
☒	☐	For Bayesian analysis, information on the choice of priors and Markov chain Monte Carlo settings
☒	☐	For hierarchical and complex designs, identification of the appropriate level for tests and full reporting of outcomes
☒	☐	Estimates of effect sizes (e.g. Cohen's *d*, Pearson's *r*), indicating how they were calculated

Our web collection on statistics for biologists contains articles on many of the points above.

Software and code

Policy information about availability of computer code

Data collection	Bruker Avance III HD and Avance II NMR spectrometer, Topspin version 3.2, Topspin version 3.5
Data analysis	nmrPipe version 9.6, NMRFAM-Sparky version 1.413, CCPN version 3, numpy (python 2.7 and python 3.6) version 1.15.2, MDAnalysis version 0.20.1, gnuplot version 5.2 pl 2, CATIA (CPMG fitting, available from www.nmrbox.org), Gromacs 2019 version.

For manuscripts utilizing custom algorithms or software that are central to the research but not yet described in published literature, software must be made available to editors and reviewers. We strongly encourage code deposition in a community repository (e.g. GitHub). See the Nature Research guidelines for submitting code & software for further information.

Data

Policy information about availability of data

All manuscripts must include a data availability statement. This statement should provide the following information, where applicable:

- Accession codes, unique identifiers, or web links for publicly available datasets
- A list of figures that have associated raw data
- A description of any restrictions on data availability

All raw data used in the figures are supplied in a separate Excel sheet (source-data)
All NMR pulse programmes (sequence), MD scripts, and analysis scripts are available from the corresponding author upon request. Expression plasmids are also available from the corresponding author upon request.

Field-specific reporting

Please select the one below that is the best fit for your research. If you are not sure, read the appropriate sections before making your selection.

[x] Life sciences [ ] Behavioural & social sciences [ ] Ecological, evolutionary & environmental sciences

For a reference copy of the document with all sections, see nature.com/documents/nr-reporting-summary-flat.pdf

Life sciences study design

All studies must disclose on these points even when the disclosure is negative.

Sample size	The number of points sampled in the NMR spectra (time-domain and CPMG-domain) was decided from standard considerations e.g. measurement time and signal-to-noise. The number of CPMG frequencies used is directly available from the figures (Fig 2 and Fig S6). A detailed description of the error estimations is given in the method section. This includes both the analysis of CPMG relaxation dispersion experiments and analysis of the enzyme activity assays.
Data exclusions	No experimental data were excluded from analysis. For the MD simulations the length of the equilibration period was judged from the RMSD to the start structure (See Figure S10)
Replication	Multiple measurements were used to calculate the uncertainties for activity assay of WT HDAC8 and mutants.<br>Multiple measurements were used to estimate errors in the CPMG relaxation dispersion experiments
Randomization	Data analysis did not require randomization.
Blinding	Blinding was not relevant to this study.

Reporting for specific materials, systems and methods

We require information from authors about some types of materials, experimental systems and methods used in many studies. Here, indicate whether each material, system or method listed is relevant to your study. If you are not sure if a list item applies to your research, read the appropriate section before selecting a response.

Materials & experimental systems

n/a	Involved in the study	
[x]	[ ]	Antibodies
[x]	[ ]	Eukaryotic cell lines
[x]	[ ]	Palaeontology and archaeology
[x]	[ ]	Animals and other organisms
[x]	[ ]	Human research participants
[x]	[ ]	Clinical data
[x]	[ ]	Dual use research of concern

Methods

n/a	Involved in the study	
[x]	[ ]	ChIP-seq
[x]	[ ]	Flow cytometry
[x]	[ ]	MRI-based neuroimaging
